# Radiation therapy generates platelet-activating factor agonists

**DOI:** 10.18632/oncotarget.7878

**Published:** 2016-03-03

**Authors:** Ravi P. Sahu, Kathleen A. Harrison, Jonathan Weyerbacher, Robert C. Murphy, Raymond L. Konger, Joy Elizabeth Garrett, Helen Jan Chin-Sinex, Michael Edward Johnston, Joseph R. Dynlacht, Marc Mendonca, Kevin McMullen, Gengxin Li, Dan F. Spandau, Jeffrey B. Travers

**Affiliations:** ^1^ Department of Pharmacology and Toxicology, Boonshoft School of Medicine at Wright State University, Dayton, OH, USA; ^2^ Department of Pharmacology, University of Colorado Health Sciences Center, Aurora, CO, USA; ^3^ Department of Dermatology, Indiana University School of Medicine, Indianapolis, IN, USA; ^4^ Department of Pathology and Laboratory Medicine, Indiana University School of Medicine, Indianapolis, IN, USA; ^5^ Department of Radiation Oncology, Indiana University School of Medicine, Indianapolis, IN, USA; ^6^ Department of Biostatistics, Wright State University, Dayton, OH, USA; ^7^ The Dayton V.A. Medical Center, Dayton, OH, USA

**Keywords:** radiation therapy, oxidized glycerophosphocholines, platelet-activating factor, cyclooxygenase type 2 enzyme, antioxidants

## Abstract

Pro-oxidative stressors can suppress host immunity due to their ability to generate oxidized lipid agonists of the platelet-activating factor-receptor (PAF-R). As radiation therapy also induces reactive oxygen species, the present studies were designed to define whether ionizing radiation could generate PAF-R agonists and if these lipids could subvert host immunity. We demonstrate that radiation exposure of multiple tumor cell lines in-vitro, tumors in-vivo, and human subjects undergoing radiation therapy for skin tumors all generate PAF-R agonists. Structural characterization of radiation-induced PAF-R agonistic activity revealed PAF and multiple oxidized glycerophosphocholines that are produced non-enzymatically. In a murine melanoma tumor model, irradiation of one tumor augmented the growth of the other (non-treated) tumor in a PAF-R-dependent process blocked by a cyclooxygenase-2 inhibitor. These results indicate a novel pathway by which PAF-R agonists produced as a byproduct of radiation therapy could result in tumor treatment failure, and offer important insights into potential therapeutic strategies that could improve the overall antitumor effectiveness of radiation therapy regimens.

## INTRODUCTION

Radiotherapy (RT) is an important modality in the management of many types of cancer [1]. Treatment plans with ionizing radiation (IR) are designed by radiation oncologists to selectively kill tumor cells, and spare normal tissue. IR damages a wide variety of targets in tumor cells through direct and indirect effects, but damage to DNA and/or DNA replication/repair machinery play major roles in radiation-induced cell death [1]. One of the consequences of IR is the generation of reactive oxygen species (ROS) [2-3]. Several groups, including ours, have demonstrated suppression of host immunity in the presence of various pro-oxidative stressors through a mechanism involving the lipid mediator Platelet-activating Factor (1-alkyl-2-acetyl-glycerophosphocholine; PAF) [4-8]. Pro-oxidative stressors including aromatic hydrocarbons found in jet fuel, chemotherapeutic agents, cigarette smoke, and ultraviolet B (UVB) radiation generate ROS that can act directly on glycerophosphocholines (GPC) to produce oxidized GPC (ox-GPC) which are potent PAF-receptor (PAF-R) agonists [9-14]. Structural studies using mass spectrometry have identified more than a dozen ox-GPC, including native PAF itself [15-18]. Yet, there is evidence for hundreds of these biologically active compounds [18-19] that have by-passed the tightly controlled enzymatic process of PAF production [20]. Like native PAF, these Ox-GPCs are metabolically unstable and their half-lives in tissue/blood are measured in minutes [20-21].

The mechanisms for PAF-R-mediated immuno-suppression have been characterized using murine models [4-8]. Using UV light as the prototype, UVB irradiation of skin results in the formation of PAF/ox-GPCs [15]. These ox-GPCs act upon the mast cell PAF-R inducing its translocation to draining lymph nodes which then, through cyclooxygenase-2 (COX-2) dependent manner [22], generate regulatory T cells (Tregs) that are the effector cells for immunosuppression [8]. PAF-R agonists produced in response to UVB/standard chemotherapeutic agents can inhibit tumor immunity via PAF→COX-2→Treg process, which is blocked by antioxidants, COX-2 inhibitors, neutralization of IL-10, and depletion of Tregs [23-24].

Since IR can induce ROS, the present studies were designed to test whether IR doses typically administered in radiotherapy clinic can generate PAF-R agonists and determine their structural composition. Moreover, these studies sought to define whether IR-generated PAF-R agonists impact radiotherapy effectiveness. These studies provide the first evidence that IR induces high levels of PAF and ox-GPC PAF-R agonists that block anti-tumor immunity via systemic PAF-R signaling in a process that can be ameliorated via COX-2 inhibitors.

## RESULTS

### Ionizing radiation generates PAF-R agonists in melanoma cells in a process blocked by antioxidants

**Figure 1:**
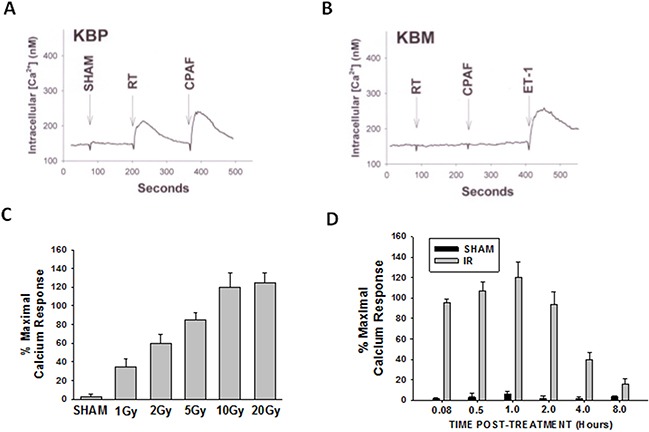
Irradiation of melanoma cells generates PAF-R agonists B16F10 cells were sham-irradiated (e.g. unirradiated) or exposed to ionizing radiation (IR; radiation therapy [RT]). **A, B.** Examples of intracellular Ca^2+^ mobilization responses in B16F10 cells that were exposed to 5 Gy IR. After 1h, lipids were extracted and PAF-R agonistic activities were measured with Ca^2+^ mobilization responses in FURA-2-labelled PAF-R-expressing KBP (A) or PAF-R-negative KBM (B) cells. KBM cells treated with 1μM endothelin 1 (ET-1) served as positive control. **C.** Dose-responsiveness of IR-induced PAF-R agonistic activity. B16F10 cells were treated with various IR doses and harvested 1h post-radiation. **D.** Time course of IR-generated PAF-R agonistic activity. B16F10 cells were irradiated with 10Gy and harvested at various times. Data are mean±SE and expressed as % maximum (1 μM CPAF) intracellular Ca^2+^ response in KBP cells from at least three separate experiments.

**Figure 2:**
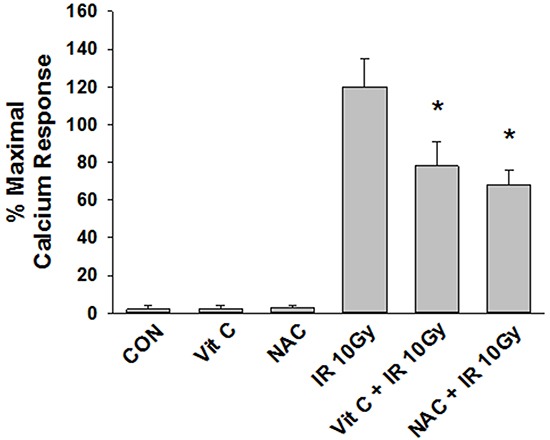
Effect of antioxidants on IR-mediated PAF-R agonists generation B16F10 cells were preincubated with antioxidants, vitamin C (2.5 mM) or N-acetyl cysteine (5 mM) for 1 h following irradiation with 10 Gy.Lipids were extracted and PAF-R agonistic activities were measured with Ca^2+^ mobilization responses in FURA-2-labelled PAF-R-expressing KBP cells. The data are the mean ± SE percentage of peak intracellular calcium response (normalized to 1 μM CPAF). * Denotes statistically significant (*P < 0.05*) fold changes from vehicle, and antioxidants alone-treated cells.

The first studies were designed to test whether IR can induce PAF-R agonist formation in melanoma cells. As multiple GPC species can act as PAF-R agonists, we quantified total PAF-R biochemical activity in lipid extracts from murine B16F10 melanoma cells following treatment with 5 Gy of IR as measured by intracellular calcium mobilization responses in Fura-2-loaded PAF-R-expressing KBP cells [7-8, 25]. Note that the amount of total PAF-R agonistic activity is defined as the Ca^2+^ mobilization peak height of normalized lipid extract as a % of the peak height from excess (1μM) of the metabolically stable PAF-R agonist carbamoyl-PAF (CPAF). Use of this semi-quantitative cellular biochemical assay allows all PAF-R activity to be measured. As shown in Figure 1A&1B, lipid extracts from B16F10 melanoma cells treated with IR induced PAF-R agonistic activity only in KBP but not in PAF-R-negative KBM cells. IR treatment generated PAF-R agonistic activity in a dose-dependent manner which appeared maximal at 10Gy (Figure 1C). Time course studies revealed that IR-generated PAF-R agonistic activity was detected almost immediately in B16F10 cells and the activity remained elevated above base-line levels at 8h post-treatment (Figure 1D). Similar to our published findings examining PAF-R biochemical activity generated in human epithelial tumor cells or human skin in response to UVB [15, 17, 26], preincubation of melanoma cells with antioxidants vitamin C or N-acetylcysteine (NAC) blocked IR-generated PAF-R agonistic activity (Figure 2). These studies indicate that IR-generated PAF-R agonistic activity in B16F10 cells are in part through a process that involves ROS.

### Structural identification of IR-generated PAF-R agonists in B16F10 cells and comparison with PAF-R agonistic activity

PAF-R agonists can be produced both enzymatically as well as via non-enzymatic free radical processes [9-11,13, 14-15, 20]. Interestingly, the majority of ox-GPCs have not been structurally characterized [16, 18-19]. To define if non-enzymatic ox-GPC PAF-R agonists are formed in response to IR, we used mass spectrometry-based structural studies using deuterium-labeled internal standards as reported [17]. In these experiments, a part of each sample was subjected to mass spectrometry and part was tested for PAF-R agonistic activity by measurement of IL-8 release in KBP cells. The advantage of the KBP-IL-8 assay is that the amount of IL-8 release can be directly compared to a dose-response curve of PAF. As shown in Table I, 1h after irradiation of B16F10 cells in-vitro with 10Gy resulted in the formation of both PAF, 1-palmitoyl 2-acetyl GPC (acyl PAF) and multiple ox-GPCs compared to sham-irradiated cells. However, no differences in the levels of the inactive PAF-precursor lyso-PAF (1-hexadecyl-2-lyso GPC) between sham-irradiated and irradiated cells were noted (Table I).

**Table I:** Effect of IR on levels of PAF and ox-GPCs in B16F10 tumor cells

Glycerophosphocholine	Control Mean (SEM)	RT Mean (SEM)
PAF 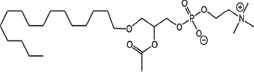	5.2 (2.9)	114(11)
PAcPC 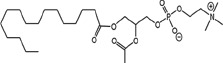	48 (18)	955(125)
BPAF 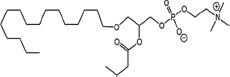	0.2 (0.2)	9.6 (2.4)
PBPC 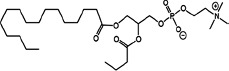	5.8 (3)	211 (43)
PPrPC 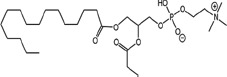	0.9 (0.5)	2.4 (0.9)
PHPC 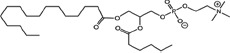	0 (0)	15.9 (7.1)
AzPAF 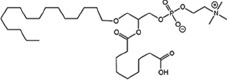	7.7 (5.6)	3.2 (3.2)
PAzPC 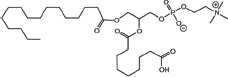	235 (148)	143 (70)
OVPAF 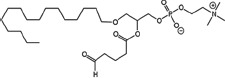	0 (0)	0 (0)
POVPC 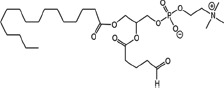	7.1 (2.0)	5.4 (0.3)
PONPC 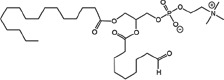	11.5 (4.2)	11.7 (0.5)
Lyso-PAF	54 (7)	56 (16)

B16F10 cells were irradiated with 10Gy or sham-irradiated in vitro. After 1 h of incubation, lipid extracts were analyzed by HPLC/MS/MS using deuterium-labeled internal standards to quantify PAF and Ox-GPC. Data are pg/10^6^ cells from at least three separate experiments. The names of each ox-GPCs in this table are: PAF, platelet-activating factor; PAcPC, 1-palmitoyl-2-acetyl-GPC; BPAF, 1-O-hexadecyl-2-butanoyl-GPC; PBPC, 1-palmitoyl-2-butanoyl-GPC; PPrPC, 1-palmitoyl-2-propanoyl-GPC; PHPC, 1-palmitoyl-2-hexanoyl-GPC; AzPAF, 1-O-hexadecyl-2-azeleoyl-GPC; PAzPC, 1-palmitoyl-2- azeleoyl-GPC; OVPAF, 1-O-hexadecyl-2-(5)oxo-valeroyl-GPC; PONPC, 1-palmitoyl-2-oxononanoyl-GPC; Lyso-PAF, inactive precursor of PAF.

**Figure 3:**
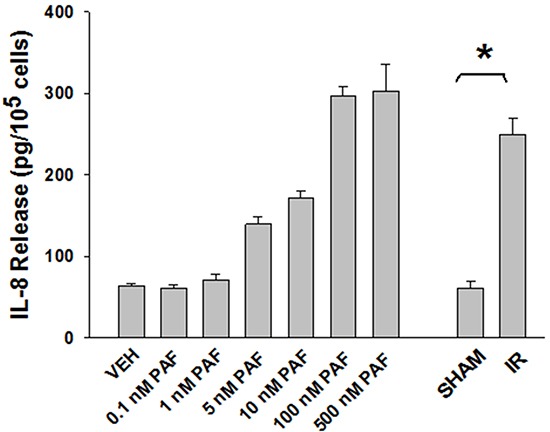
Ionizing Radiation of B16F10 cells generates PAF-R agonistic activity as measured by IL-8 production in KBP cells B16F10cells were sham-irradiated or irradiated with 10Gy IR. After 1 h of incubation, lipids were extracted and added to the culture of KBP cells. Following 6 h of incubation, supernatant was removed and IL-8 release was measured by ELISA to test for the PAF-R agonistic activity. In addition, KBP cells were incubated with various doses of PAF or vehicle and used as positive and negative controls. This experiment was performed in triplicate and repeated three times. * Denotes statistically significant (*P < 0.05*) changes from vehicle or sham-treated cells.

Approximately 1/10^th^ of the lipid extracts were tested for PAF-R agonistic activity by incubation with KBP cells and IL-8 measured in the supernatant 6h later by ELISA as a surrogate for PAF-R activation [27]. As shown in Figure 3, PAF induced IL-8 formation in KBP cells in a dose-dependent manner. There was basal PAF-R agonistic activity in lipid extracts derived from unirradiated (sham) B16F10 cells similar to that observed in cells treated with vehicle and at lower doses of PAF (Figure 3). However, high levels of IL-8 were measured in the supernatants of KBP cells treated with lipid extracts taken from B16F10 cells exposed to IR (Figure 3). As expected, these lipid extracts did not generate increased levels of IL-8 release in KBM cells (*data not shown*). When these lipid extract-derived IL-8 PAF-R activities were compared with known PAF, we estimated the complex mixture of GPC in the lipid extracts contained the equivalent of 409 pg of PAF per 10^6^ cells. However, mass spectrometry studies indicate that ∼110pg of PAF per 10^6^ cells were found in the lipid extracts (Table I). These studies suggest that a significant amount of the IR-induced PAF-R agonistic activity resides in non-PAF ox-GPCs.

### Ionizing radiation generates PAF-R agonists in multiple tumor cell types in-vitro and in-vivo

**Figure 4:**
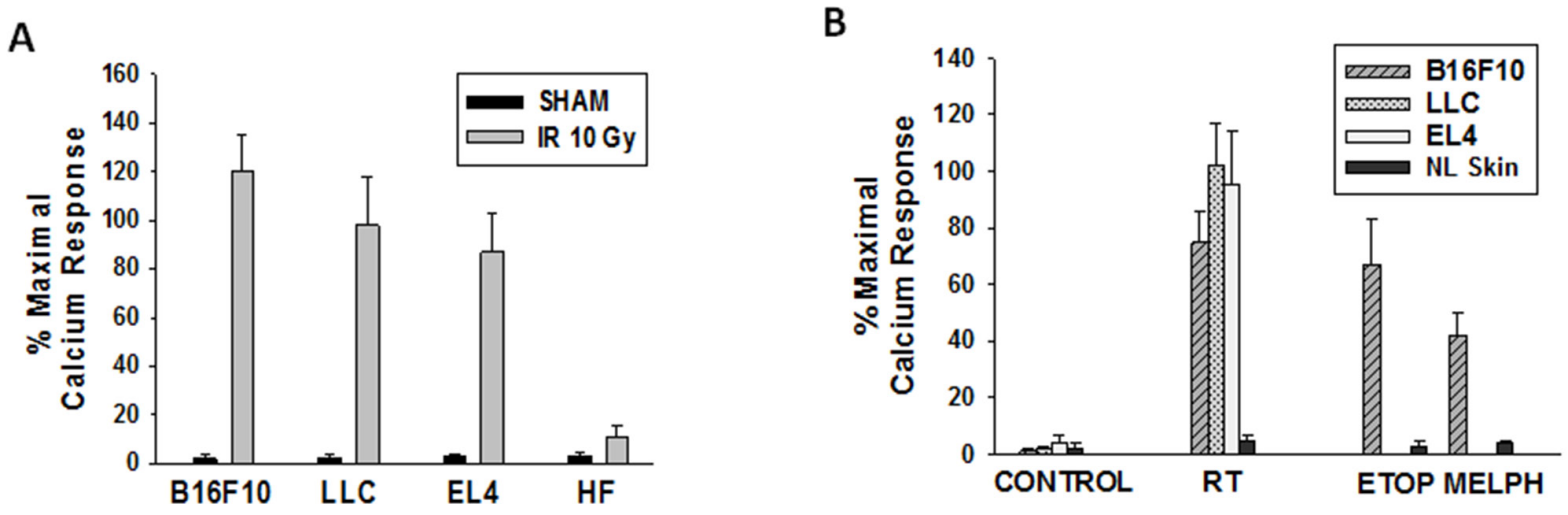
Effects of IR on PAF-R agonists production from several tumor types **A.** Murine B16F10, LLC1, EL4 and human fibroblasts were treated with sham or 10Gy IR. **B.** WT mice were implanted with B16F10, LLC1, EL4 cells (0.5×10^6^) into both hindlimbs. Once tumors reached 10mm size, one tumor was treated with 5Gy IR or etoposide (ETOP; 36mg/kg) or melphalan (MELPH; 15mg/kg) and other tumor with sham or vehicle (100μl PBS). Following 1h incubation (A&B) cells and tumors harvested and lipids extracts were tested for PAF-R agonist activity. Human fibroblasts (in-vitro) and normal skin (in-vivo) treated ± IR or chemotherapeutic agents were used as controls. The data are mean±SE percentage of peak calcium response (normalized to CPAF) from 3 separate experiments (A) and 4-6 separate tumors (B).

The next studies were designed to test if IR can generate PAF-R agonists in other tumor types both in-vitro and in-vivo. For these studies we used tumor types syngeneic to C57BL/6 mice. As shown in Figure 4A, treatment of Lewis lung carcinoma (LLC1) or T-cell lymphoma (EL4) cells generated significant levels of PAF-R agonistic activity. Of note, irradiation of primary cultures of human fibroblasts yielded very little PAF-R agonistic activity (Figure 4A). To define if radiotherapy of tumors in-vivo generated PAF-R agonists, we implanted 5 × 10^5^ B16F10, LLC1, or EL4 tumor cells into both hind limbs of C57BL/6 mice. When the tumors reached approximately 10mm diameter, the tumor on one limb was treated with 5Gy, while the other tumor was shielded from IR (left untreated). One hour following irradiation, the mice were euthanized, and the tumors dissected, weighed, and lipid extract assayed for PAF-R agonistic activity using our KBP Ca^2+^ assay. In some experiments, normal non-tumor-bearing skin was treated with a similar dose of IR and used as a control. As shown in Figure 4B, radiation exposure resulted in an increased level of PAF-R agonistic activity in various tumor types. However, IR of normal skin resulted in only negligible PAF-R agonistic activity.

We have reported that intratumoral chemotherapy of B16F10 tumors also generates PAF-R agonistic activity [24]. To compare the levels of PAF-R agonists generated between intratumoral chemotherapy and IR, we subjected mice previously implanted with two B16F10 tumors to intratumoral injections of etoposide (36mg/kg) or melphalan (15mg/kg) to one tumor with the other tumor treated with 50 μl of saline vehicle [24]. After 1h, the tumors were harvested and lipid extracts tested for PAF-R agonistic activity as outlined above. As depicted in Figure 4B, the levels of PAF-R agonistic activity generated by intratumoral chemotherapy resembled those tumors treated with IR. Again, subcutaneous injection of chemotherapy into normal skin did not generate appreciable amounts of PAF-R agonistic activity (Figure 4B). These studies indicate that IR generates PAF-R agonists in diverse tumor types but not in normal skin.

### PAF-R activation diminishes effectiveness of IR in a COX-2-dependent process

**Figure 5:**
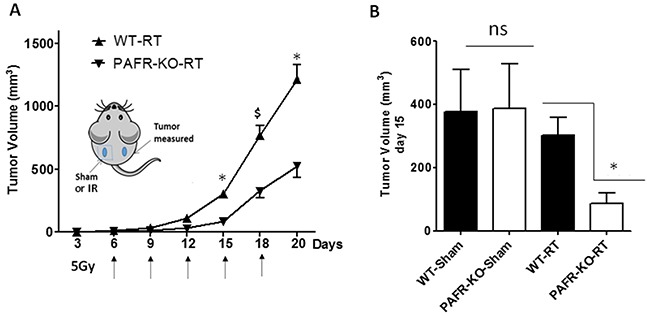
Localized irradiation augments the growth of untreated B16F10 melanomas in a PAF-R-dependent manner **A.** Groups of 6-7WT and *Ptafr−/−* (PAFR-KO) mice were implanted with PAF-R deficient B16F10 tumors on both the dorsal hind flanks (day 0). Six days later (and q 2-3days afterwards) left side tumors were sham-irradiated or irradiated with 5Gy of IR. The right side tumors left untreated (shielded). Tumor growth was measured over time and tumor volume was calculated. The data are mean±SE of tumor volume of untreated tumors. **B.** Tumor volume of untreated tumors at day 15 from sham and IR-treatment. There were significant differences in the growth of RT-treated *(*P<0.05*) or $ (*P <0.1*) but not sham-treated tumors (ns= non-significant). This experiment was repeated with additional 6-7 mice/group with similar results.

**Figure 6:**
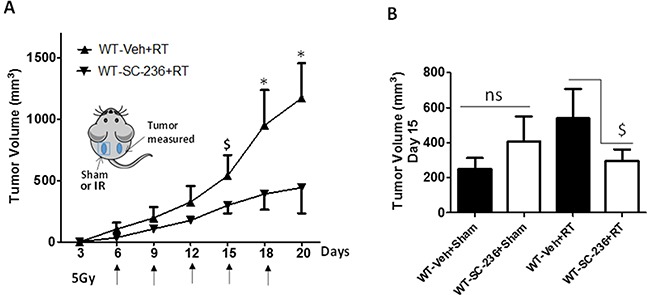
Role of COX-2 in RT-mediated augmentation of tumor growth **A.** Groups of 6-13 WT mice were implanted with PAF-R deficient B16F10 tumors on both the dorsal hind flanks (day 0). Following tumor implantation (day 0) and every 3 days mice were i.p. injected with or without COX-2 inhibitor, SC-236 (200 ng). After 6 days of tumor implantation when mice developed palpable tumors (10mm), left side tumors were irradiated with 5Gy of IR and repeated at every q2-3 days and right side tumors left untreated. Tumor growth was assessed over time and tumor volume was calculated. **B.** A representative tumor volume data at day 15 (right side) in all the experimental groups. The data depicted are the mean ± SE of tumor volume of untreated tumors over time. Statistical significant differences were noted in the growth of RT-treated *(*P<0.05*) or $ (*P<0.1*) but not sham-treated tumors (ns denotes non-significant).

Given our previous findings that intratumoral chemotherapy of one tumor modulates the growth of a second tumor in a PAF-R-dependent fashion in a process involving Tregs and COX-2 metabolites [24], we tested if irradiation of one tumor can modulate the growth of a second tumor. Our protocol was modified in that WT and PAF-R-deficient (*Ptafr−/−*, PAFR-KO) mice underwent implantation with B16F10 tumors on both dorsal hindquarters (2 tumors/mouse). The left flank tumors were either sham-irradiated or irradiated with 5Gy starting at day 6 of tumor implantation and repeated every 2-3 days until the termination of the experiment, while the other (right flank) tumors left undisturbed (shielded). Irradiation of one tumor resulted in an enhanced growth of the second (undisturbed) tumor in WT compared with *Ptafr−/−* hosts (Figure 5). Though irradiation resulted in growth inhibition of the treated left flank tumors, loss of host PAF-R function exerted no perceptible effect on the left flank tumor growth characteristics (Supplementary Figure S1). To assess whether COX-2 is crucial for PAF-R mediated augmentation of tumor growth, WT mice were implanted with two B16F10 tumors and then treated with intraperitoneal injections of the COX-2 inhibitor (SC-236) or saline vehicle starting at day 0 and every 3 days afterwards. As shown in Figure 6, the COX-2 inhibitor blocked the tumor growth enhancing effects of IR on the second tumor. We did not note any effects of COX-2 inhibitor exposure on the irradiated (left) tumor (*data not shown*). These studies reveal that IR generates adequate levels of PAF-R agonists to augment tumor growth. Moreover, this process was attenuated by pharmacologic inhibition of COX-2.

### Identification of PAF-R agonists produced in response to radiotherapy of cutaneous tumors in humans

To test whether IR exposure results in PAF-R agonists production in humans, we identified subjects who were receiving radiation treatments for different types of tumors. Following informed consent, a 2 mm punch biopsy was obtained from a tumor before RT. Immediately following the RT procedure, a second 2 mm punch biopsy was obtained from the tumor. The biopsies were subjected to lipid extraction and 1/10^th^ of the extracts tested for PAF-R agonistic activity via our KBP Ca^2+^ assay, with the remainder subjected to mass spectrometry to quantify PAF and other structurally known GPCs. As shown in Table II, all of the four irradiated tumor samples contained PAF-R agonistic activity, which ranged from 2-24X baseline levels. Structural characterization of the samples using mass spectrometry identified elevated levels of PAF and several ox-GPC species. Clinical pictures of two of the subjects, a large basal cell carcinoma and a bladder carcinoma, are found in Supplementary Figure S2. These studies indicate that clinical RT exposure can generate PAF-R agonists in human tumors.

**Table II:** Identification of PAF and ox-GPCs in human skin following RT

Tumor Type	Treatment	PAF	PAcPC	BPAF	PBPC	GPAF	PHPC	AzPAF	PAzPC	OVPAF	ONPAF	POVPC	PONPC	Ca^2+^ Response
BCC #1	Pre-RT	3216	83	53	48	170	ND	6615	28255	7	6	6	15	25%
	**RT (0.3 Gy)**	**4583**	**242**	**79**	**163**	**482**	**ND**	**10345**	**50059**	**27**	**16**	**7**	**64**	**78%**
BCC #2	Pre-RT	2633	45	30	30	109	ND	3623	18631	3	8	6	27	30%
	**RT (0.3 Gy)**	**5950**	**59**	**117**	**203**	**396**	**4**	**9713**	**39834**	**12**	**36**	**9**	**82**	**62%**
Bladder Ca	Pre-RT	2504	140	36	35	98	1	2991	15623	10	9	3	27	8%
	**RT (0.7 Gy)**	**7410**	**376**	**205**	**309**	**534**	**5**	**10240**	**43593**	**15**	**37**	**7**	**69**	**22%**
Pseudo-	Pre-RT	23	72	15	23	2	7	424	NM	26	280	NM	1328	2%
lymphoma	**RT (0.2 Gy)**	**3086**	**1031**	**96**	**127**	**25**	**28**	**1663**	**NM**	**103**	**832**	**NM**	**4511**	**48%**

Values of PAF and other GPC are pg per 90% of lipid extracts from 2 mm punch biopsy specimen; Ca^2+^ response denotes % of peak Ca^2+^ mobilization response compared to 1 μM CPAF in FURA-2-loaded KBP cells from 5% of the biopsy specimen. The names of each ox-GPCs in this table are: PAF, platelet-activating factor; PAcPC, 1-palmitoyl-2-acetyl-GPC; BPAF, 1-O-hexadecyl-2-butanoyl-GPC; PBPC, 1-palmitoyl-2-butanoyl-GPC; GPAF, 1-O-hexadecyl-2-glutaroyl-GPC; PHPC, 1-palmitoyl-2-hexanoyl-GPC; AzPAF, 1-O-hexadecyl-2-azeleoyl-GPC; PAzPC, 1-palmitoyl-2- azeleoyl-GPC; OVPAF, 1-O-hexadecyl-2-(5)oxo-valeroyl-GPC; ONPAF, 1-O-hexadecyl-2-oxononanoyl-GPC; POVPC, 1-palmitoyl-2-(5)oxo-valeroyl-GPC; PONPC, 1-palmitoyl-2-oxononanoyl-GPC.

## DISCUSSION

While radiotherapy is commonly used to treat human malignancies, it is not considered a good treatment option as a single agent for malignant melanoma [1]. The present studies describe a previously unappreciated mechanism by which IR exposure results in immunosuppression [28-29]. These existing data support the model that PAF and ox-GPC PAF-R agonists produced in part due to ROS from IR can exert systemic immunosuppressive effects.

Oxidation of esterified fatty acyl residues introduces oxy functions, rearranges bonds and fragments carbon-carbon bonds by ɷ-scission that generate a myriad of phospholipid reaction products including PAF-R agonists [14]. In contrast to tightly controlled enzymatic pathways for PAF biosynthesis, large amounts of numerous ox-GPC PAF-R agonists can be produced non-enzymatically. Though this has been an area of intensive study for multiple groups including ours, only a small number of the structures of these ox-GPCs have been elucidated [17, 24]. The present studies not only demonstrate that IR-generated PAF-R agonistic activity is diminished by antioxidants, but structural characterization of this activity reveals ox-GPCs known to be produced non-enzymatically. By comparison of PAF produced versus non-PAF agonistic activity (Figure 3), it appears that majority of IR generated PAF-R agonists are due to ox-GPCs, not enzymatically produced PAF. These findings are consistent with IR being a potent pro-oxidative stressor. It is likely that tumor cellular membranes serve as the source of PAF and oxidized phospholipids from the IR and are thus the source of RT-mediated PAF-R agonist formation in the experimental murine and human models used. This finding is supported by the negligible production of PAF-R agonistic activity found in normal murine skin following RT. However, it appears that IR does result in at least some PAF-R agonist formation in human skin, as has been reported using a rodent model of IR of skin window chambers and very sensitive PAF-R assay of leukocyte adhesion in the presence/absence of a PAF-R antagonists [45].

Radiotherapy has been demonstrated to exert an immunostimulatory effect in both preclinical as well as human studies [30-32]. This “abscopal effect” is well-known to radiation biologists and consists of shrinkage of other tumors which are not in the radiation field during RT. The mechanisms for this abscopal effect and why it is seen only in some cases and not others are not entirely clear. It is possible that the generation of PAF-R agonists as a by-product of RT could provide an explanation for why RT-mediated abscopal effects are not seen consistently in the clinic. It should be noted that in our experimental conditions we did not observe an abscopal effect. Interestingly, our previous murine models have suggested that only certain tumors (B16F10, LLC1), but not others (EL4) are affected by exogenous PAF agonists [23-24, 33-34]. Moreover, PAF and ox-GPC PAF-R agonist accumulation can be affected by multiple factors. These include diet, as Western diet with high levels of arachidonic acid can provide high levels of PAF-R agonist precursor 1-alkyl GPC species with highly unsaturated fatty acids at the *sn-2* position. In addition, these bioactive lipids can be affected by levels of the PAF- and ox-GPC metabolizing enzyme serum PAF-acetylhydrolase (PLA2G7). Of note, there are both genetic and acquired deficiencies of this enzyme which could possibly play an important role in RT clinical responses [35].

Tumor resistance to therapeutic treatments such as RT and chemotherapy is an important clinical problem and is an area of active study. In contrast to cellular resistance to the effects of these agents, the present studies using RT and our previously reported ones using chemotherapy and UVB describe a novel mechanism by which these modalities could subvert anti-tumor immunity. Indeed, our studies using UVB and chemotherapy provide evidence implicating anti-tumor immunity, in particular Tregs in PAF-mediated effects on B16F10 tumor growth [23-24]. First, PAF effects are not seen when tumors are placed in immunodeficient mice [23]. Second, use of PAF-R-negative B16F10 cells transduced with functional PAF-Rs implanted in WT vs *Ptafr−/−* hosts have confirmed that the PAF-R mediating the response is on the host, not tumor. Finally, use of neutralizing antibodies against IL-10 or depleting Tregs both block PAF-mediated augmentation of experimental tumor growth. Our findings fit with the report that high doses of IR in murine tumor models of RT generate high levels of Tregs, a finding not seen after exposure to low doses of IR [36].

Exogenous pro-oxidative stressors ranging from aromatic hydrocarbons to cigarette smoke to UVB radiation have been shown to induce systemic immunosuppression via PAF-R signaling which is blocked by antioxidants [5, 7-8, 10]. Apoptotic cells generate PAF and contribute to melanoma tumor progression via PAF-R activation [37]. The production of PAF-R agonists from these various agents begins a cascade of events leading to systemic immunosuppression. The cytokines which appear to be critical for the immunosuppression include IL-10 and COX-2-generated eicosanoids [4, 6, 8]. Mast cells and Tregs are also implicated in PAF-R-dependent systemic immunosuppression [8, 22, 38]. The present studies demonstrating that COX-2 inhibitor blocks IR-mediated augmentation of experimental tumor growth are not only consistent with previous studies characterizing the role of this eicosanoid-generating enzyme in PAF-mediated systemic immunosuppression [4, 6, 8] they also provide the rationale for future studies testing the ability of COX-2 inhibitors to enhance the effectiveness of RT. It should be noted that COX-2 inhibitors have been shown to exert not only radioprotective properties on the host, but can also serve as radiosensitizing agents [39-40]. Moreover, COX-2 inhibitors also exert direct antitumor effects in a variety of tumor types [41-42].

In summary, the present studies provide the first evidence that IR can generate PAF and that PAF-R signaling can inhibit RT effectiveness. In contrast to resistance mechanisms that are applicable at the tumor cell level, this process is likely due to the subversion of host tumor immunity. That RT generates PAF-R agonists in humans is suggestive that this novel pathway could have tremendous clinical significance. Since this process involves the pro-oxidative qualities of RT and is potentially neutralized by antioxidants, with downstream effects susceptible to COX-2 inhibition, these studies could provide the impetus for future studies to define the clinical significance of this novel pathway in humans.

## MATERIALS AND METHODS

### Reagents and cell lines

All chemicals were obtained from Sigma-Aldrich (St. Louis, MO) unless indicated otherwise. Murine melanoma B16F10, lung carcinoma Lewis Lung Carcinoma (LLC1) and T cell lymphoma cell line EL4 cells were obtained from ATCC (Boston, MA) were grown in DMEM high glucose media with 10% FCS as described [23-24, 33-34]. PAF-R-negative KB cells were rendered PAF-R-positive (KBP) by transducing the MSCV2.1 retrovirus encoding the human leukocyte PAF-R and PAF-R-deficient (KBM) by transducing with the vector alone and grown in DMEM high glucose media with supplements as described previously [25]. Primary cultures of human fibroblasts were obtained from neonatal discarded foreskins and grown as described [43]. Cell lines were grown to approximately 80-90% confluence in 10 cm dishes, and washed three times with Hanks Balanced Salt Solution (HBSS) and then incubated with 2 ml of pre-warmed (37°C) HBSS with 10mg/ml fatty acid-free BSA with 2 μM of the serine hydrolase inhibitor pefabloc. B16F10 melanoma cells were radiated with multiple doses of ionizing radiation and incubated at various time points as given in the figure legends. Cell irradiation studies were conducted using a 160-kVp Faxitron X-ray machine. Settings for the machine were as follows: 0.5 mm Al filter, d = 33 cm, and dose rate of 251.7 cGy/min. In some experiments, antioxidants were preincubated for 60 min before addition of chemotherapeutic agents or DMSO (0.5%) vehicle. The incubations were quenched by addition of 2 ml of ice-cold methanol followed by methylene chloride, and lipids extracted as described [7-8].

### Mice

Female C57BL/6-wild type mice (PAF-R expressing; age 6-8 week) were purchased from The Charles River Laboratories. Age-matched female PAF-R-deficient (*Ptafr−/−*) mice on a C57BL/6 background, generated as described [44], were a kind gift of Professor Takao Shimizu (Department of Biochemistry, University of Tokyo). All mice were housed under specific pathogen-free conditions and all procedures were approved by the Institutional Animal Care and Use Committee of Indiana University School of Medicine (IUSM).

### Measurement of PAF-R agonists

#### Calcium mobilization studies

The presence of systemic PAF-R agonists in lipid extracts derived from IR and chemotherapeutic agent-treated tumors/cell lines was measured by the ability of lipid extracts to induce an intracellular Ca^2+^ mobilization response in PAF-R-expressing KBP cells, but not in PAF-R-deficient KBM cells as described [7-8, 25].

#### PAF-R IL-8 release

PAF-R agonistic activity in lipid extracts was also measured by measuring IL-8 released into the supernatants of KBP vs KBM cells as reported [8].

#### Mass spectrometry studies

Mass spectrometry was performed on cell lines and perfusion samples using the AB Sciex (Foster City, CA) triple quadrupole QTRAP® 5500 mass spectrometer, equipped with a CTC-PAL autosampler and a Shimadzu HPLC as described [17]. Please see Supplementary Methods for details.

#### In-vivo tumor PAF production and growth studies

To determine the ability of RT to produce PAF-R agonists, 5×10^5^ B16F10, LLC1 or EL-4 cells (which lack functional PAF-R [23-24, 33-34]), were implanted subcutaneously into both hind flanks of WT and *Ptafr−/−* mice to produce two tumors followed by treatment with or without x-ray. In some studies tumors were injected with 50μl of either etoposide (36mg/kg), melphalan (15mg/kg), or PBS with 0.5%DMSO vehicle. The mice were anesthetized at 1h post-irradiation and tumors dissected, weighed, and subjected to lipid extraction.

To determine the ability of IR to modulate tumor growth, B16F10 cells were implanted into both hindlimbs and the mice were subjected to IR q3 days starting at day 6. To define the ability of COX-2 inhibitors to modulate IR-mediated tumor growth, SC-236 (200ng) or 100μl PBS were injected i.p. at day 0 and every 3 days afterwards [24]. When tumors became approximately 10mm in largest dimension, then one tumor was treated with radiation, while the other tumor was out of the x-ray field and was also shielded with lead. Tumors were irradiated with 5 Gy of x-rays using a Precision XRAD 320 (North Branford, CT) (250 kVP, dose rate of 1.442 Gy/min, 2mm Aluminum filter, 50 cm from source). Tumors were treated with x-rays 5 different times, with 2-3 days between treatments. Tumor growth (major circumference and minor circumference) was monitored and measured at various times with digital calipers (Mitituyo), and tumor volume was calculated (major circumference × minor circumference^2^/2).

#### Human RT studies

Subjects undergoing localized RT for tumors in skin and metastasized to the skin (from bladder cancer patient) as part of their management were recruited for these studies. Following informed consent, a 2mm punch biopsy was obtained from the tumor. Following RT (within 5-10min after treatment), the subject then underwent a 2mm punch biopsy from the treated tumor. The 2mm punch biopsies were placed immediately in ice-cold methanol and lipids extracted using our standard protocol [7]. 1/10^th^ of the lipid extract was tested for PAF-R agonistic activity using calcium mobilization responses in KBP cells compared to CPAF [24]. The rest of the lipid extracts were subjected to mass spectrometry [17]. The human studies were approved by IUSM Institutional Review Boards.

#### Statistical analysis

For all murine studies, individual experiments were performed using at least 6 mice per group and repeated as necessary (at least twice) to verify reproducibility and provide additional data for analysis. All statistical calculations were performed using SAS Version 9.3 and R Version 3.2.0. For murine studies, the analysis focused at the end of the study (days 15-20), where available. For murine and in-vitro data, we used 2 sample t-tests to compare two groups at different time points. The normality of data and equal variances were checked by Shapiro-Wilk's and Levene's tests and was a reasonable assumption in all cases. To further explore the treatment effect during the whole study period and time, we used 2 way analysis of variance test. The data represent mean±SE values. Differences were considered statistically significant when *P*<0.05 and marginally significant when *P<*0.10.

## SUPPLEMENTARY MATERIALS AND METHODS FIGURES



## Abbreviations

COX-2: cyclooxygenase type 2
CPAF: 1-hexadecyl-2-N-methylcarbamoyl glycerophosphocholine
GPC: glycerophosphocholine
ox-GPC: oxidized GPC
IL-10: interleukin 10
IR: ionizing radiation
NAC: N-acetylcysteine
PAF: platelet-activating factor
PAF-R: PAF receptor
PAF-AH: PAF acetylhydrolase
RT: radiation therapy
ROS: reactive oxygen species
Treg: regulatory T cells
