# Supplementary material for: Radiation therapy generates platelet-activating factor agonists
Source: Oncotarget. 2016 Mar 3;7(15):20788–800. doi: 10.18632/oncotarget.7878 (PMC4991492; doi:10.18632/oncotarget.7878)
Supplement: Supplementary file 1 [file oncotarget-07-20788-s001.pdf]

SUPPLEMENTARY MATERIALS AND METHODS

Mass spectrometry analysis

After PDT versus sham treatment of HaCaT cells, lipids were extracted from 4-6 x 10^7 cells using the Bligh and Dyer method. Five nanograms of 1-O-hexadecyl-2-[2,2,2-$^2H_3$]acetoyl-*sn*-glycero-3-phosphocholine (d_3-PAF) were added during the extractions as internal standard as well as five nanograms each of deuterated retention time reference standards d_3-PAcPC, d_3-18ePAF, and d_3-SAcPC. Oxidized phosphatidylcholines (Ox-GPC) were isolated from the majority of other lipids, as described (Kayganich-Harrison KA, Murphy RC. Anal Biochem, 1994; 221(1):16-24.; Harrison KA, *et al.* J Mass Spectrom, 2000; 35(2):224-236.). Butylated hydroxy toluene was added (50 µL of 10 µM) to the 4 mL methanol:water (4:1) fractions to prevent further oxidation during shipping or storage. Samples were shipped overnight on dry ice and stored at -70 °C until evaporated to dryness/near dryness under vacuum in a Savant Speed Vac Plus, SC110A at low heat setting. Residues were transferred with two 100 µl methanol rinses to 1 ml conical glass HPLC injection vials, concentrated again, then diluted to 100 µl with 20% aqueous methanol and crimp-capped. 20% of the samples were injected for HPLC/MS/MS analysis.

Standard curves for Platelet-activating factor (PAF), butyroyl PAF and Azoleoyl PAF analogs, using d_3PAF as internal standard were linear ($R^2 > 0.999$) using HPLC/MS/MS in multiple reaction monitoring mode. Standard curves using *sn*1-heptadecenoyl-*sn*2-lyso GPC (17:1aLPC) and *sn*1-O-(d_4)hexadecyl-*sn*2-lyso GPC (d_4-lysoPAF) were also constructed to estimate the quantites of the lysoGPCs relative to the d_3-PAF internal standard. Samples were analyzed using the AB Sciex (Foster City, CA) triple quadrupole QTRAP® 5500 mass spectrometer, equipped with a CTC-PAL autosampler and a Shimadzu HPLC. HPLC separation utilized a 2.1x100 mm Kinetex C18 (2.6u, 100 A) column with a 2mm Security Guard pre-column (Phenomenex, Torrance, CA). Mobile phase consisted of (solvent A) methanol, acetonitrile, water 6:2:2 (v/v/v) and 5:5:2 methanol, acetonitrile, dicholormethane (solvent B) both with 2 mM ammonium acetate. The gradient employed was 20% B, held for 1.5 min, to 75% B in 22 min, then ramped up to 99% B in 0.1 min then held for 18 min to elute non-oxidized GPCs in the sample. The column eluate, 0.22 mL/min, was transferred directly into the electrospray ion source, with the ion spray voltage of -4200 V to generate negative ions, nebulizing gas flow set at 20 and turbospray gas flow set to 10 at 200 °C. The declustering potential (DP) was set to -10 V when monitoring collision induced dissociation (CID) reactions of $[M-H]^-$ ions or $[M+acetate]^-$ adduct ions and to -220 V for monitoring CID reactions of $[M-CH_3]^-$ ions from the GPC species. During multiple reaction monitoring (MRM) the collision energy was -68 V for collision induced dissociation (CID) of $[M+acetate]^-$ adduct ions, -52 V for CID of $[M-CH_3]^-$ ions, and -38 V for CID of [M-H]- ions. The dwell time of each MRM transition was 20 ms, with a two second duty cycle. MRM transition pairs for species previously identified as PAF receptor agonists monitored included 1-O-hexadecyl-2-acetoyl-GPC (16e 2:0; PAF), 1-O-hexadecyl-2-butanoyl-GPC (16e 4:0; BPAF), 1-O-hexadecyl-2-(5)oxo-valeroyl-GPC (16e 5al; OVPAF), 1-O-hexadecyl-2-glutaroyl-GPC (16e 5COOH; GPAF), 1-O-hexadecyl-2-oxononanoyl-GPC (16e 9al; ONPAF), 1-O-hexadecyl-2-azeleoyl-GPC (16e 9COOH; AzPAF), 1-palmitoyl-2-acetyl-GPC (16a 2:0; PAcPC), 1-palmitoyl-2-propanoyl-GPC (16a 3:0; PPrPC) 1-palmitoyl-2-butanoyl-GPC (16a 4:0; PBPC), 1-palmitoyl-2-(5)oxo-valeroyl-GPC (16a 5al; POVPC), 1-palmitoyl-2-glutaroyl-GPC (16a 5COOH; PGPC), 1-palmitoyl-2-hexanoyl-GPC (16a 6:0; PHPC), 1-palmitoyl-2-oxononanoyl-GPC (16a 9al; PONPC), and 1-palmitoyl-2- azeleoyl-GPC (16a 9COOH; PAzPC). The transitions monitored to measure 1-O-acyl-lyso GPCs, 1-O-alkyl lyso GPCs and 1-O-alk-1'-enyl lyso GPCs (plasmenyl LPC) were of the [M+acetate] ions to the specific fragment ions seen for each type of lyso-GPC subspecies; carboxylate anion, 1-O-alkyl-glycero-phosphate ion via neutral loss of choline acetate, or alk-1'-enyl-oxy anion, respectively. See reference (Kayganich-Harrison KA, Murphy RC. Anal Biochem, 1994; 221(1):16-24.) for a complete listing of MRM transitions monitored. Reference standards were not available for every species that was monitored. Quantitation of these species was done by comparison to the standard curve constructed from a reference standard of a similar species. CID mass spectra of all these species were obtained in concurrent or separate analyses in order to confirm identities of the species to be quantified. Corroborating data for these identifications was obtained by relative retention times to known reference standards and ionization behavior. For instance, aldehyde containing GPC species are known to form a hemiacetal adduct with the alcohols in the mobile phase during negative ion ESI, resulting in $[M+OAc + MeOH]^-$ ions. Also, GPC species containing a dicarboxylic acid, such as AzPAF, form $[M-H]^-$ ions rather than $[M-CH_3]^-$. Furthermore, fragmentation of $[M-H]^-$ ions from dicarboxylic acid-containing GPC species transfer a methyl group (14 Da) from the choline to the dicarboxylic acid group upon CID fragmentation. Optimal declustering potentials and collision energies of the different ions and adducts and CID fragmentations

were determined using reference standards, if available, or oxidized GPC species produced in-house by oxidation of linoleate- or arachidonate-containing GPC species. For most of the GPC species MRM transitions were monitored for both the $[M+acetate]^-$ and the $[M-CH_3]^-$ ions, to provide additional confirmation of identity. The MRM transition monitored for aldehyde-containing GPCs also included that of the $[M+32Da+acetate]^-$ ion. The MRM transition monitored for ω-carboxyl-containing GPCs was that of the $[M-H]^-$ ion to the $[RCOO+14Da]^-$ ion (methyl transfer).

Negative enhanced product ion (EPI) scans were obtained concurrently with the MRM analysis for some species. The scan rate was 10000 Da/s, with dynamic fill time enabled for the LIT (range 0.05-100 ms). The DP and CE were the same values as for the corresponding MRM transition pair for the species being monitored.

SUPPLEMENTARY FIGURES

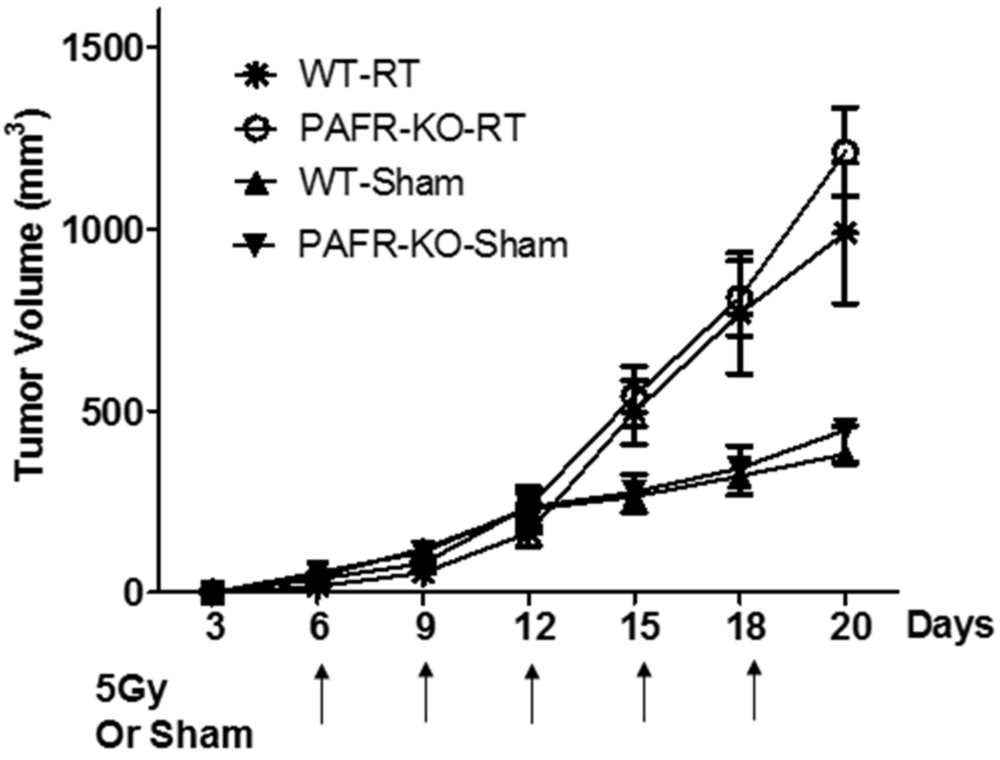

Supplementary Figure S1: Effects of localized IR treatment on the growth of melanoma tumors in WT and PAFR-KO mice. WT and PAFR-KO (*Ptafr*-/-) mice (5-7 mice per group) harboring PAF-R deficient B16F10 tumors sham-irradiated or irradiated with 5 Gy starting day 6 and repeated q3days afterwards. Tumor growth was measured (major circumference and minor circumference) over time and tumor volume was calculated (major circumference x minor circumference^2/2). The data depicted are the mean ± SE of tumor volume of sham versus irradiated (RT) tumors over time.

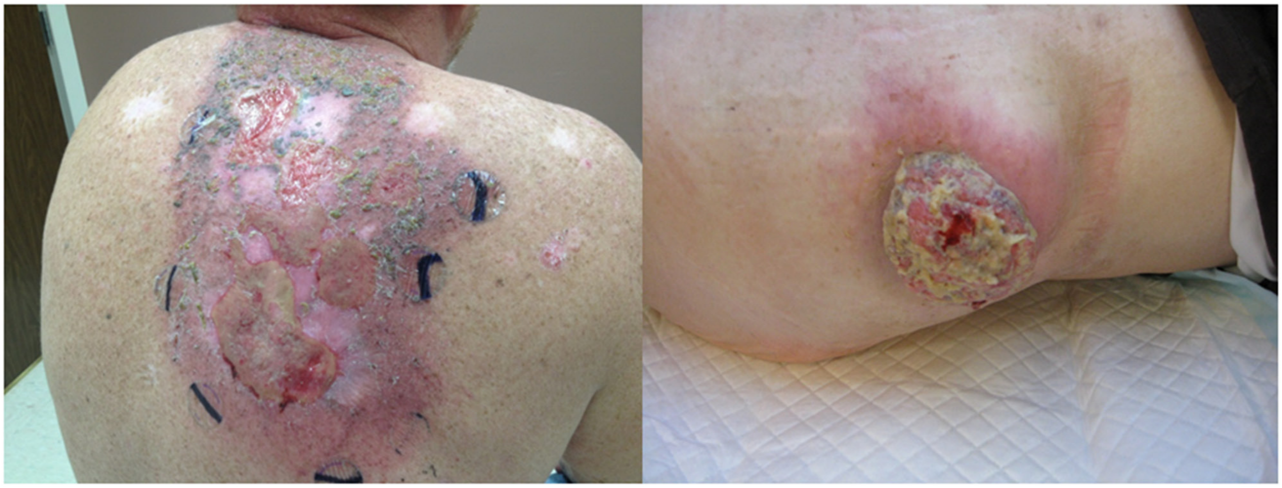

Supplementary Figure S2: Examples of subjects undergoing radiation therapy. The picture on the left is a large basal cell carcinoma on the back of Subject #1; the right is a biopsy-proven metastatic bladder carcinoma on the abdomen of Subject #3. See Table II for details.
